# Identification and quantification of lipid accumulation in adipose tissue using oil red O and Sudan stains

**DOI:** 10.6026/9732063002001663

**Published:** 2024-11-05

**Authors:** Swati Shrivastava, Aishwarya Srivastava, Ruchi Jain, Amit Kumar Srivastava, Sourabh Shrivastava, Rashmi Sathe

**Affiliations:** 1 Department of Biochemistry, Government Medical College, Datia, Madhya Pradesh, India; 2 Department of Biochemistry, All India Institute of Medical Sciences, Gorakhpur, Uttar Pradesh, India; 3 Department of Anatomy, MLB Medical College, Jhansi, Uttar Pradesh, India; 4 Department of Anatomy, Government Medical College Datia, Madhya Pradesh, India; 5 Department of Anaesthesiology, GAJRA Raja Medical College, Gwalior, Madhya Pradesh, India; 6 Department of Oral Medicine and Radiology, People's College of Dental Sciences and Research Centre, Bhopal, Madhya Pradesh, India

**Keywords:** Oil Red O, Sudan stains, adipose tissue, obesity, oral diseases

## Abstract

This study investigates the application of Oil Red O and Sudan stains in identifying and quantifying lipid accumulation in adipose tissue, specifically focusing on its relevance to obesity-related oral diseases. Adipose tissue samples were collected from 50 obese patients (BMI > 30) and 50 normal-weight controls. Samples were stained with Oil Red O and Sudan III, IV and Black. Lipid accumulation was quantified using digital image analysis. Oral health examinations assessed the prevalence of periodontal disease, dental caries and oral candidiasis. Oil Red O staining show 2.8-fold higher lipid contents obese subjects than controls (p<0.001). Sudan stains demonstrated similar trends, with Sudan Black being the most sensitive (3.2-fold increase, p<0.001). Positive correlations were found between lipid accumulation and the severity of periodontal disease (r=0.72, p<0.001), dental caries (r=0.58, p<0.01) and oral candidiasis (r=0.63, p<0.01) in obese subjects. Oil Red O and Sudan stains effectively identify and quantify lipid accumulation in adipose tissue. The study's findings underscore a robust link between increased lipid content and the prevalence of obesity-related oral diseases, highlighting the potential of these staining techniques in oral health research and clinical practice.

## Background:

The prevalence of obesity has reached epidemic proportions, impacting a vast number of people globally and presenting substantial health hazards [1]. Obesity is now widely acknowledged as a significant risk factor for the development of several oral disorders, such as periodontal disease, dental caries and oral candidiasis [2- 3]. The intrinsic processes connecting obesity to oral health issues are intricate and diverse, with the buildup of lipids in adipose tissue playing a leading role [4]. The adipose tissue, previously seen as a passive organ for storing energy, is now acknowledged as an active endocrine organ that releases a range of bioactive substances, such as adipokines and pro-inflammatory steroids [5]. In obesity, the excessive buildup of lipids in adipose tissue results in persistent low-grade inflammation and modified immunological responses, which can significantly impact dental health [6-7]. Accurate detection and quantification of lipid accumulation in adipose tissue are crucial for gaining a deeper understanding of the contribution of obesity to oral illnesses. Histological staining methods, specifically Oil Red O and Sudan stains, are now recognized as useful instruments for viewing and examining the lipid composition in tissue samples [8- 9]. The detection of neutral lipids is commonly achieved using Oil Red O. However, various lipids can be visualized using Sudan stains such as Sudan III, IV and Black [10]. This study aims to examine the use of Oil Red O and Sudan stains to detect and measure lipid production in adipose tissue samples obtained from obese persons. Additionally, the study seeks to examine the relationship between lipid content and the occurrence of oral disorders associated with obesity. Our objective is to clarify these connections to enhance our knowledge of the development of oral disorders in obese individuals and maybe pinpoint novel areas for preventive and therapeutic approaches [11- 12].

## Methods and Materials:

## Study population:

The study comprises of 100 adult participants aged between 18 and 65 years, recruited from the Department of Dentistry. The research group consisted of fifty obese adults with a body mass index (BMI) of 30 kg/m^2^ or more, whereas 50 normal-weight individuals with a BMI of 18.5-24.9 kg/m^2^ acted as controls. The exclusion criteria encompassed pregnancy, systemic disorders impacting lipid metabolism and the use of drugs recognized to interact with lipid levels. The institutional ethics committee authorized the study protocol and all subjects gave written informed permission.

## Acquisition of adipose tissue samples:

Each participant's abdomen region was analyzed using a 6 mm punch biopsy instrument to collect subcutaneous adipose tissue samples, each measuring approximately 1 cm^3^ and while under local anesthesia. Specimens were promptly immersed in a 10% neutral buffered formalin solution for 24 hours. Samples of fixed adipose tissue were subjected to conventional histological procedures for processing and staining. The specimens were enveloped in an optimal cutting temperature (OCT) compound and subjected to freezing at a temperature of -80°C. Cryosections with a thickness of 8 micrometres were generated using a Leica CM1950 cryostat from Leica Bio systems. Following a 30-minute air-drying period, sections were fixed in a 4% paraformaldehyde solution for 10 minutes and then stained with a freshly produced working solution of Oil Red O (0.5% in propylene glycol) for 30 minutes at room temperature. The slides were counterstained with hematoxylin for 30 seconds. The Sudan staining procedure was conducted using Sudan III, Sudan IV and Sudan Black B solutions. The sections were immersed in a 4% paraformaldehyde solution, washed with 70% ethanol and then stained with either 0.7% Sudan III in 70% ethanol, 0.7% Sudan IV in 70% ethanol, or 0.3% Sudan Black B in 70% ethanol for a duration of 15 minutes. The slides underwent counterstaining with hematoxylin. Visual analysis and quantification were performed on stained sections using a light microscope (Olympus BX53) with a digital camera (Olympus DP74). Ten randomly selected fields were recorded from each sample at a magnification of 400 x. Image J software (NIH, USA) analyzed images to measure the proportion of area that showed positive staining for lipids. The analyst performed picture analysis without knowledge of the group assignment.

## Oral Health Examination:

Each participant had a thorough oral health examination performed by two professionally trained dentists. The examination comprised:

[1] Periodontal evaluation: The depth of probing, the level of clinical attachment and the occurrence of bleeding during probing were documented at six locations per tooth. Periodontal disease severity was classified according to the CDC-AAP case definition.

[2] The DMFT (Decayed, Missing and Filled Teeth) index evaluated the dental caries state.

[3] Oral candidiasis screening involves examining the oral mucosal surfaces for indications of candidiasis.

[4] The presence of Suspicious lesions were verified by fungal culture.

The statistical data analysis was conducted using SPSS version 25.0, developed by IBM Corp. in Armonk, NY, USA. Statistical normality of the data distribution was evaluated by the Shapiro-Wilk test. Appropriate statistical tests, such as Student's t-test or Mann-Whitney U test, were used to analyze the differences in lipid buildup between the obese and control groups. Pearson's or Spearman's correlation coefficients were calculated to assess the relationships between lipid accumulation and oral health markers. A statistical significance level was defined as a p-value less than 0.05.

## Results:

## Lipid accumulation in adipose tissue:

The application of Oil Red O and Sudan stains revealed significant differences in lipid accumulation between obese subjects and normal-weight controls. Table 1 summarizes the quantitative analysis of lipid content in adipose tissue samples.

Sudan Black B demonstrated the highest sensitivity in detecting lipid accumulation, showing a 3.2-fold increase in stained area in obese subjects compared to controls. Oil Red O, Sudan III and Sudan IV also showed significant increases in lipid content in the obese group, with 2.8-fold, 2.6-fold and 2.7-fold increases, respectively.

## Oral health status:

The prevalence and severity of oral health issues were markedly higher in the obese group compared to the control group. Table 2 presents the oral health parameters for both groups.

## Correlation between lipid accumulation and oral health:

Strong positive correlations were observed between lipid accumulation in adipose tissue and the severity of oral health issues in the obese group. Table 3 shows the correlation coefficients between lipid content (as measured by Sudan Black B staining) and oral health parameters.

These results demonstrate a strong association between increased lipid accumulation in adipose tissue and the prevalence and severity of obesity-related oral diseases. The study highlights the effectiveness of Oil Red O and Sudan stains in quantifying lipid content and their potential utility in investigating the relationship between obesity and oral health.

## Discussion:

The reported 2.8-fold rise in lipid content measured by Oil Red O staining in obese individuals is consistent with prior research that have documented significant lipid build up in obesity [13]. Sudan stains, namely Sudan Black B, exhibited much greater sensitivity, indicating their potential superiority in identifying a wider variety of lipids in adipose tissue [14]. The results emphasize the usefulness of histological staining methods in obesity research and their possible usability in clinical environments. The robust positive correlation (r=0.72) between lipid accumulation and the severity of periodontal disease provides more support for the increasing body of research that associations obesity with periodontal health [15]. An explanation for this correlation may be attributed to the pro-inflammatory condition caused by an excessive amount of adipose tissue, which results in heightened synthesis of cytokines and adipokines that can worsen periodontal inflammation [16]. Moreover, insulin resistance associated with obesity can hinder the processes of tissue healing, therefore exacerbating periodontal disease [17]. Further inquiry is warranted by the fascinating discovery of a connection (r=0.58) between lipid content and dental caries. Although the precise mechanism by which adipose tissue lipids trigger caries is not completely understood, it is likely to be influenced by the systemic consequences of obesity on salivary composition, changes in the oral flora, or dietary patterns linked to excessive sugar consumption [18-19]. The correlation between lipid accumulation and the frequency of oral candidiasis (r=0.63) contributes to the increasing evidence of modified host defence mechanisms in an obese population [20]. Hypertrophy of adipose tissue might impair immunological function, therefore heightening vulnerability to opportunistic fungal infections such as candidiasis [21]. Subsequent investigations should examine the long-term impacts of weight reduction on the lipid composition of adipose tissue and the implications for mouth health. Furthermore, exploring the molecular processes that connect malfunction of adipose tissue to oral illnesses should offer novel targets for therapeutic approaches [22]. The present work underscores the efficacy of Oil Red O and Sudan stains in the quantification of lipid accumulation in adipose tissue and establishes a robust correlation between elevated lipid levels and oral illnesses associated with obesity. The significance of systemic elements, namely malfunction of adipose tissue, in the development of oral illnesses is underscored by these results. Moreover, the findings indicate that the treatment of obesity might have a vital role in the prevention and control of oral health problems in those who are overweight or obese [23]. This work demonstrates that the use of histological staining techniques provides new opportunities for research in oral health problems associated to obesity. Potential clinical uses of this technology may include risk assessment and monitoring of oral illnesses associated to obesity. Additional investigations are required to clarify the intricate relationship among adipose tissue, systemic inflammation and oral health, which may result in innovative preventative and therapeutic approaches in dental treatment for individuals with obesity [24- 25].

## Conclusion:

The present work provides evidence for the efficacy of Oil Red O and Sudan stains in the quantification of lipid accumulation in adipose tissue samples obtained from both obese and normal-weight people. Our results demonstrate a notable rise in lipid concentration in adipose tissue of individuals with obesity, with Sudan Black B exhibiting the greatest sensitivity among the staining techniques employed. Furthermore, we have identified robust positive associations between the lipid content of adipose tissue and the occurrence and intensity of oral illnesses associated to obesity, such as periodontal disease, dental caries and oral candidiasis. These findings highlight the intricate connection between obesity and oral health, indicating that malfunction of adipose tissue may be a key factor in the development of dental illnesses in obese persons.

## Figures and Tables

**Table 1:** Lipid content in adipose tissue samples (% of stained area)

**Staining Method**	**Obese Group (n=50)**	**Control Group (n=50)**	**p-value**
Oil Red O	78.3 ± 6.2	28.1 ± 4.7	<0.001
Sudan III	82.5 ± 5.8	31.7 ± 5.2	<0.001
Sudan IV	80.9 ± 6.5	30.4 ± 4.9	<0.001
Sudan Black B	89.7 ± 4.3	28.1 ± 5.5	<0.001
Values are presented as mean ± standard deviation.

**Table 2:** Oral health parameters in obese and control groups

**Parameter**	**Obese Group (n=50)**	**Control Group (n=50)**	**p-value**
**Periodontal Disease Severity**			
- Mild	12 (24%)	35 (70%)	<0.001
- Moderate	23 (46%)	12 (24%)	<0.001
- Severe	15 (30%)	3 (6%)	<0.001
DMFT Score	14.7 ± 4.2	6.3 ± 2.8	<0.001
Oral Candidiasis Prevalence	18 (36%)	4 (8%)	<0.001
Values are presented as n (%) or mean ± standard deviation.

**Table 3:** Correlation between lipid accumulation and oral health parameters in obese group

**Oral Health Parameter**	**Correlation Coefficient (r)**	**p-value**
Periodontal Disease Severity	0.72	<0.001
DMFT Score	0.58	<0.01
Oral Candidiasis Presence	0.63	<0.01
